# Sustainable Boron Nitride Nanosheet-Reinforced Cellulose Nanofiber Composite Film with Oxygen Barrier without the Cost of Color and Cytotoxicity

**DOI:** 10.3390/polym10050501

**Published:** 2018-05-05

**Authors:** Hoang-Linh Nguyen, Zahid Hanif, Seul-A Park, Bong Gill Choi, Thang Hong Tran, Dong Soo Hwang, Jeyoung Park, Sung Yeon Hwang, Dongyeop X. Oh

**Affiliations:** 1 Research Center for Bio-based chemistry, Korea Research Institute of Chemical Technology (KRICT), Ulsan 44429, Korea; nguyen@krict.re.kr (H.-L.N.); zahid@krict.re.kr (Z.H.); seula@krict.re.kr (S.-A.P.); thangth@krict.re.kr (T.H.T.); 2 Division of Environmental Science & Engineering, Pohang University of Science and Technology (POSTECH), Pohang 37673, Korea; dshwang@postech.ac.kr; 3 Department of Chemical Engineering, Kangwon National University, Ganwan-do, Samcheok 25913, Korea; bgchoi@kangwon.ac.kr; 4 Advanced Materials and Chemical Engineering, University of Science and Technology (UST), Daejeon 34113, Korea

**Keywords:** cellulose nanofiber, boron nitride nanosheet, oxygen barrier, food packaging

## Abstract

This paper introduces a boron nitride nanosheet (BNNS)-reinforced cellulose nanofiber (CNF) film as a sustainable oxygen barrier film that can potentially be applied in food packaging. Most commodity plastics are oxygen-permeable. CNF exhibits an ideal oxygen transmission rate (OTR) of <1 cc/m^2^/day in highly controlled conditions. A CNF film typically fabricated by the air drying of a CNF aqueous solution reveals an OTR of 19.08 cc/m^2^/day. The addition of 0–5 wt % BNNS to the CNF dispersion before drying results in a composite film with highly improved OTR of 4.7 cc/m^2^/day, which is sufficient for meat and cheese packaging. BNNS as a 2D nanomaterial increases the pathway of oxygen gas and reduces the chances of pinhole formation during film fabrication involving water drying. In addition, BNNS improves the mechanical properties of the CNF films (Young’s modulus and tensile strength) without significant elongation reductions, probably due to the good miscibility of CNF and BNNS in the aqueous solution. Addition of BNNS also produces negligible color change, which is important for film aesthetics. An in vitro cell experiment was performed to reveal the low cytotoxicity of the CNF/BNNS composite. This composite film has great potential as a sustainable high-performance food-packaging material.

## 1. Introduction

Synthetic polymers such as polyethylene (PE), polypropylene (PP), and polyethylene terephthalate (PET) have been widely used as food- and medicine-packaging materials owing to their high strength, low cost, viscoelastic properties and chemical resistance. The oxygen barrier properties of food and medicine packaging films are vital to prevent the oxidation of food and medicine. However, most polymeric films are oxygen permeable and exhibit a high oxygen transmission rate (OTR) of 40–1000 cc/m^2^/day [1,2,3,4,5].

Halogenated and metalized polymeric films can achieve an OTR of 0.1–10 cc/m^2^/day [6,7,8,9]. However, these polymer films pose many environmental and health threats [6,7,8,9]. For example, the incineration of aluminum-coated PET films and polyvinylidene chloride (PVDC) produces fine dust and dioxins, respectively. In general, metalized polymeric films are not recyclable.

Cellulose nanofiber (CNF) is a sustainable and biocompatible nanomaterial [10] and is a potential food- and medicine-packaging material [11,12,13,14,15,16,17,18,19]. It is produced by mechanically disintegrating highly crystalline nanofibrils in cellulose bulk, the most abundant biomass [10,20]. Coatings and films composed of CNF can achieve an OTR of less than 1 cc/m^2^/day, which is suitable for the packaging of most foods and medicines [11,12,13,14,15,16,17,18,19].

It is questionable whether the oxygen barrier performance of CNFs can be reproduced in an industrial setting. CNF films are typically produced from aqueous dispersion by air drying, and the resultant films often exhibit high OTR values (19.08 cc/m^2^/day, by our measurement), because the capillary force during drying results in a heterogeneous surface [20]. The OTR value is as high as that of bare PET.

Two-dimensional (2D) nanomaterials, including graphene and MoS_2_, have been introduced in polymeric films and shown to improve the oxygen barrier properties of the resulting matrix films, because the layered structure increases the pathway distance for oxygen gas [21,22,23,24]. Graphene and MoS_2_ are colored and have high optical absorption coefficients [25,26]. Their addition to milky or transparent CNF films decreases the esthetic value of the packaging material produced. In addition, graphene is cytotoxic [27]; these disadvantages limit the food-packaging applications of 2D nanomaterials. 

Boron nitride nanosheet (BNNS) is a 2D nanomaterial having several advantages as a filler for food-packaging films. It can be produced on a larger scale with lower costs than graphene [28,29]. In addition, it is white-colored and known to be less cytotoxic than the 2D nanomaterials considered previously [30].

The combination of CNF and BNNS is uncommon [24,31], but recently some studies have reported that BNNS/CNF composite films exhibit good thermal conductivity [32,33,34,35,36]. However, these studies of BNNS/CNF composites did not examine their gas-barrier performance.

**Scheme 1:**
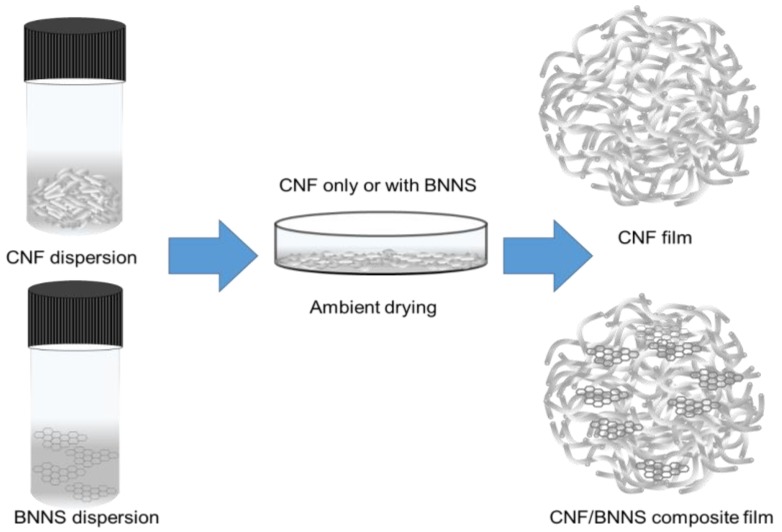
A brief procedure to prepare pristine and boron nitride nanosheet (BNNS)-containing cellulose nanofiber (CNF) films.

In this study, we show that a BNNS-containing CNF composite film can achieve a low OTR of ~4.7 cc/m^2^/day (Scheme 1). The film was prepared by simple air drying of the corresponding aqueous solution. Thus, this method is easily scalable for the production of CNF-based barrier films and is based on a sustainable aqueous system. Typically, PP, PE and PET films exhibit the OTR values of >1000, >1000, and 10–100 cc/m^2^/day, respectively [1,2,3,4,5,13]. The oxygen-barrier performance of the prepared BNNS/CNF composite film is suitable for use as a packaging film for meats and cheese [13]. The BNNS addition had minimal effect on the optical properties of the CNF film and improved the tensile strength by a factor of ~1.23 without a significant elongation reduction. In addition, the composite film did not show any cytotoxicity to a cell line.

## 2. Materials and Methods

### 2.1. Materials

Boron nitride (BN) and an ionic liquid ([EMIM][BF_4_]) were purchased from Sigma Aldrich (USA). A ~3 wt % CNF aqueous dispersion was purchased from the University of Maine (Orono, ME, USA). The width and length of the CNF were ~50 nm and several μm, respectively.

### 2.2. Boron Nitride Nanosheet (BNNS) Synthesis

BNNS was synthesized as described in a previous report [36]. Briefly, BN was exfoliated and functionalized in a Taylor–Couette (TC) reactor composed of two concentric inner and outer stainless-steel cylinders with a radius ratio of η = R_i_/R_o_ = 0.92 and an aspect ratio of Γ = L/d = 2.3, where R_i_ is the inner cylinder radius, R_o_ is the outer cylinder radius, L is the cylinder length, and d is the outer cylinder diameter. BN powder was dispersed in a solution of deionized (DI) water/ionic liquid [EMIM][BF_4_] (0.15 vol %). The feed solution (30 mg/mL) was injected into the TC reactor and allowed to react for 1 h. After this process, the resultant dispersion samples were centrifuged (420× *g*, 150 min) to remove unbounded [EMIM][BF_4_] and un-exfoliated BN sheets. The BNNS powder samples were collected by freeze-drying for 24 h. The concentrations of BNNS in water can be adjusted up to 10 mg/mL by mild sonication of BNNS powders in water.

### 2.3. Composite Film Preparation

Pristine CNF and CNF/BNNS nanocomposite films were prepared as follows (Scheme 1). BNNS solution (1.79 mg/mL) was dropped onto a CNF solution (0.5 wt %) under stirring, and the resultant suspension was subsequently mixed at 12,000 rpm using a high-speed stirrer (Ultraturrax T25, IKA, San Diego, CA, USA) for 10 min, followed by degassing with an ultrasonic cleaner (SD-D400H, lklab Co., Namyangju, Korea) for 20 min. A total of 100 g of the suspension was then poured into a polystyrene petri dish (150 mm in diameter) and dried under ambient conditions for 6 days. The films were peeled from the petri dish and stored in a desiccator prior to further characterization.

### 2.4. Tensile Properties

The tensile tests of the films were performed with a universal testing machine (Instron 5943, Instron Corp., Norwood, MA, USA) with a 1000 N load cell. The films were cut into a dog-bone shape. The test area of the samples was ~26.5 mm in length, ~3.2 mm in width (Figure S1), and ~70.0 μm thick. The tests were performed at a strain rate of 1 mm/min under ambient conditions. A total of three specimens were tested for each type of sample. To interpret the data of the tensile properties, the statics and methods need to consider (Supplementary Materials).

### 2.5. Oxygen Transmission Rate (OTR)

The OTRs of the composite films at different loadings of BNNS were measured with an automated oxygen-permeability testing machine (Lyssy L100-5000, Systech Instruments Ltd., Thame, UK). The test area of the samples was 65 cm^2^ and the tests were performed at 23 °C and 50% relative humidity using high purity oxygen gas (99.999%) following the ASTM D3985 standard protocol.

### 2.6. Characterization

The light transmittance spectra of the films were measured from 400–800 nm with a ultraviolet/visible (UV-vis) spectrophotometer (UV-2600, Shimadzu, Kyoto, Japan). The structure and morphology of the prepared nanocomposite films were characterized using a field emission scanning electron microscope (FE-SEM, MIRA 3 XMU, TESCAN, Brno, Czech Republic) equipped with the OIMTM-technology from EDAX/TSL, operating at an acceleration voltage of 10 kV. The FE-SEM samples were prepared by vacuum sputtering Pt onto the dried sample under ambient conditions. Transmission electron microscopy (TEM) images were obtained using an E.M. 912 Ω energy-filtering TEM (EF TEM 120 kV) and a JEM-3010 HR TEM (300 kV). A scanning transmission electron microscope (STEM) was operated with a probe focused to 0.2 nm and a camera length of 20 cm. The scan raster was 512 × 512 points with a dwell time of 8.5 s per scan.

### 2.7. Cytotoxicity Test

The cell viability test was performed on the surfaces of pristine and 5 wt % BNNS-containing composite CNF films [37,38,39]. The fully swollen film disks with the same diameter as the 24-well plate were immersed in ethanol for 12 h and washed with phosphate-buffered saline (PBS) just before cell seeding. A mouse pre-osteoblast cell line, MC3T3-E1, was cultured in minimal essential medium-alpha (MEM-α; Hyclone, Cramlington, UK) supplemented with 10% fetal bovine serum (FBS; Hyclone, Cramlington, UK) and 1% penicillin/streptomycin (Hyclone, Cramlington, UK) at 37 °C under a humidified atmosphere of 5% CO_2_ and 95% air. The subconfluent cells were detached using 0.25% trypsin-EDTA (Hyclone, Cramlington, UK), and the viable cells were counted using the trypan blue assay. The cells were further seeded onto film-containing and empty wells as a control in a 24-well plate at a density of approximately 3 × 10^4^ cells per well and cultured for 3 days. The number of viable cells as a function of culture time (0 to 3 days) was determined via a colorimetric assay (CCK-8, Dojindo, Santa Clara, CA, USA); the number of viable cells is proportional to the light absorbance value at 450 nm.

## 3. Results and Discussion

### 3.1. Appearance of Cellulose Nanofiber (CNF) and BNNS Solutions

**Figure 1:**
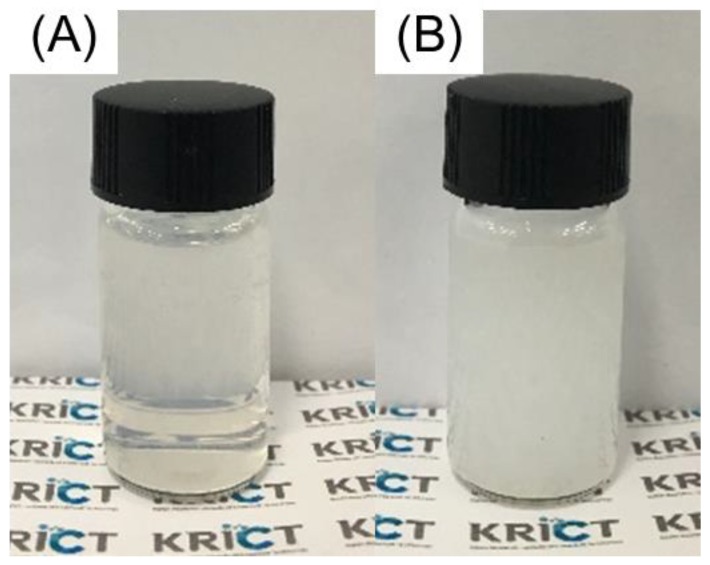
Apparent features of the (**A**) BNNS and (**B**) CNF aqueous dispersions.

The CNF and BNNS aqueous dispersions are opaque and translucent, respectively, and both are white-colored (Figure 1). The similar color of the BNNS filler and CNF film is beneficial for esthetic reasons. The CNF and BNNS particles did not precipitate even after 1 year, indicating that both particles were stably dispersed in the aqueous solutions. Other types of cellulose nanomaterials with surface charges, such as cellulose nanocrystals and carboxylated CNF, are transparent [20]. However, CNF has no surface charge and its fibers were partially aggregated, resulting in an opaque dispersion [20].

### 3.2. Analysis of BNNS Particles

**Figure 2:**
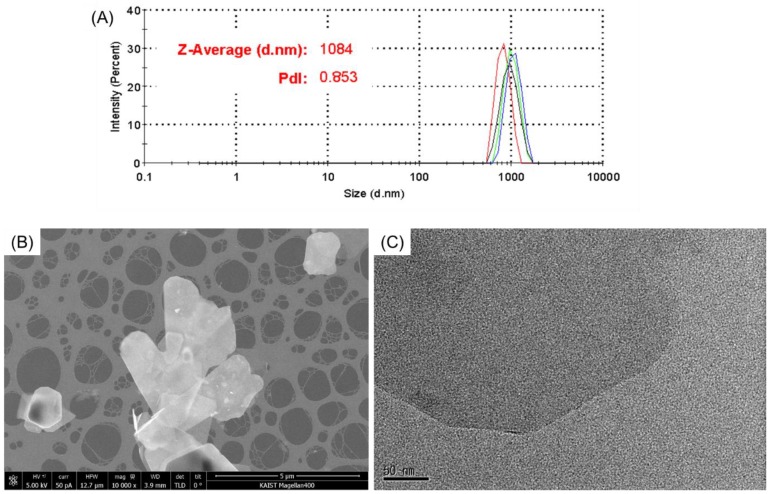
(**A**) Zeta sizer analysis of the synthesized BNNS particle solution with Z-average size and polydispersity (PDI) values; (**B**) Scanning electron microscope (SEM) and (**C**) transmission electron microscope (TEM) images of the synthesized BNNS particles. Scale bars are 5 μm and 50 nm, respectively.

BNNS was prepared as a nanometer-thick sheet. The dimensions of the synthesized BNNS particles were investigated using a zeta sizer, SEM, and TEM (Figure 2). The zeta average size and polydispersity (PDI) of the BNNS particles were ~1084 nm and ~0.85, respectively (Figure 2A). The particle size is represented as the hydrodynamic diameter of an equivalent sphere. Thus, the zeta average size would be similar to the longest length of the nanosheets. The relatively low PDI suggests that the TC reactor-based exfoliation produced uniformly sized BNNS particles. The >1000 nm length of BNNS is the main reason that its aqueous solutions are translucent.

In the SEM image (Figure 2B), ~1 μm sized BNNS particles were observed. The length of the BNNS particles determined from the SEM images is compatible to the zeta average size. The TEM image shows a more magnified shape of a single BNNS particle (Figure 2C). In the TEM image, the BNNS is translucent, indicating that the electron beam was transmitted through the BNNS particle, likely because of its nm thickness.

### 3.3. Preparation of BNNS-Containing CNF Composites

Pristine CNF and BNNS/CNF composite films with the different BNNS contents of 0–5 wt % were prepared by drying the corresponding aqueous dispersions (Scheme 1). Because both CNF and BNNS were prepared as aqueous dispersions, homogenous BNNS/CNF composite films were obtained. Ionic liquids are not volatile [40,41,42], so during the preparation of the pristine CNF and composite films, mostly water evaporated.

### 3.4. Optical Properties of the BNNS-Containing CNF Composite

**Figure 3:**
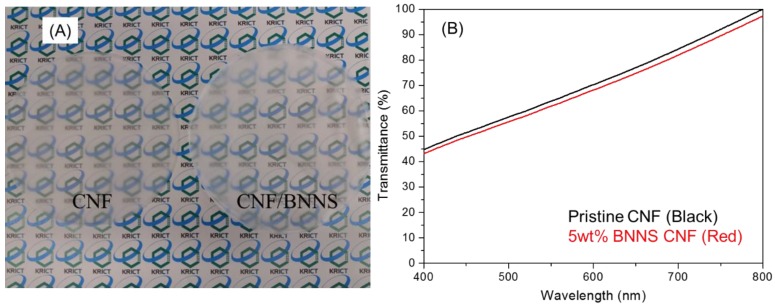
(**A**) Picture and (**B**) ultraviolet/visible (UV-vis) transmittance of the pristine and 5 wt % BNNS-containing CNF films.

The resultant pristine CNF and BNNS/CNF composite films were white and translucent, respectively (Figure 3A). Both pristine CNF and 5 wt % BNNS-containing CNF composite films show similar light-transmittance patterns, and a transmittance of only several percent was observed at 400–800 nm (Figure 3B). The 5 wt % BNNS addition exhibited only minimal effects on the light transmittance of the CNF film, and the color change was also negligible upon the addition of BNNS.

### 3.5. Morphology of the BNNS-Containing CNF Composite

**Figure 4:**
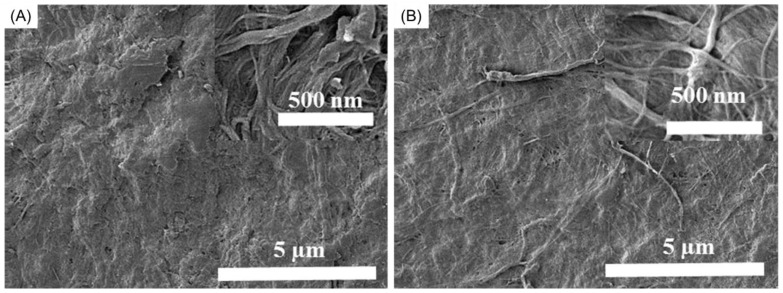
SEM images of (**A**) pristine and (**B**) 5 wt % BNNS-containing CNF films.

The surface morphology of the pristine CNF and BNNS/CNF composite films was investigated by SEM (Figure 4). Figure 4A shows the typical surface morphology of a CNF film where the nanofibril structure can be observed. The SEM image of the 5 wt % BNNS-containing CNF film exhibited an analogous morphology, indicating that the BNNS addition did not significantly affect the surface morphology of the CNF film. In addition, BNNS particles were not observed on the surface of the 5 wt % BNNS-containing CNF film (Figure 4B).

### 3.6. Oxygen Transmission Rate of BNNS-containing CNF Composite

**Figure 5:**
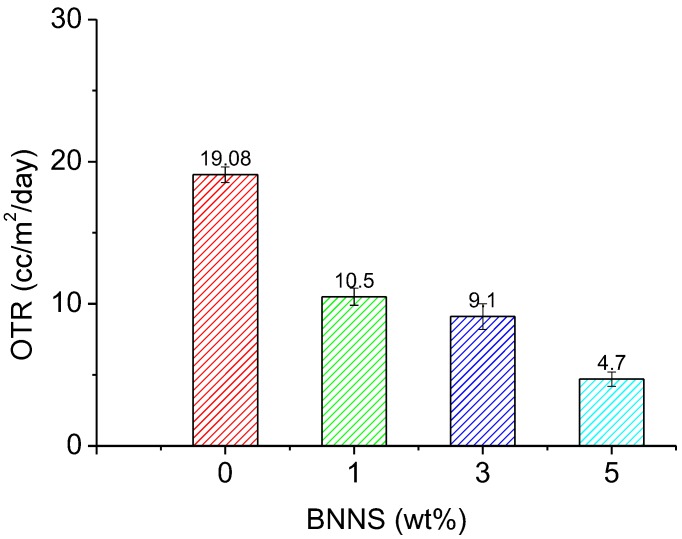
Oxygen transmission rate (OTR) of pristine and BNNS-containing CNF films. The data of triplicate samples represent mean ± deviation.

As observed in previously reported 2D nanomaterials, BNNS addition improved the oxygen barrier properties of the CNF film (Figure 5). The pristine CNF film exhibited an OTR of 19.08 cc/m^2^/day, which is similar to that of the bare PET film [1,2,3,4,5]. The barrier performance of the pristine CNF is not sufficient for use in most food-packaging applications. As previously mentioned, CNF films typically exhibit an OTR of <1 cc/m^2^/day under ideal conditions. However, during large-scale production of CNF films, the capillary force induced by water drying can result in pinholes in the film [20]. The OTR values of the BNNS-containing CNF composites gradually decreased with increasing BNNS content to 4.7 cc/m^2^/day. The oxygen-barrier performance of the composite film is similar to that of ethylene vinyl alcohol (EVOH), a typical oxygen-barrier polymeric film, and is suitable for use as a packaging film for meat and cheese [13]. Nevertheless, the OTR can still be improved to <1 cc/m^2^/day, which would be similar to metalized PET and PVDC films that are used in most food-packaging applications.

### 3.7. Tensile Properties of the BNNS-Containing CNF Composite

**Figure 6:**
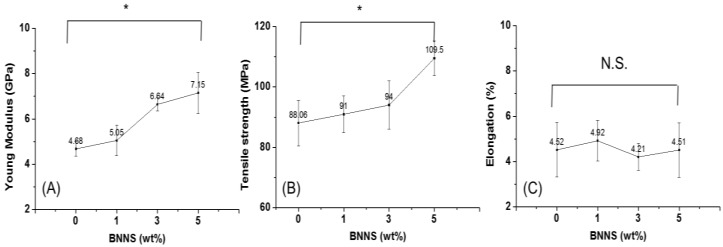
Tensile properties of pristine and BNNS-containing CNF films: (**A**) Young’s modulus; (**B**) tensile strength; and (**C**) elongation. The data of triplicate samples represent mean ± deviation. Statistical significance of a comparison of 0 wt % and 5 wt % BNNS-containing samples (N.S., not significant, *p* > 0.05; *, *p* < 0.05; unpaired *t*-test). The data statics is described in the Supplementary Materials.

The 2D geometry of the BNNS enhanced the mechanical properties of the composite films. The tensile tests of the pure and BNNS-containing CNF films provided quantitative measures of their Young’s moduli, tensile strengths and elongations (Figure 6, Figures S2–S5). Young’s modulus, tensile strength, and elongation of the pristine CNF were ~4.7 ± 0.3 GPa, ~88.1 ± 7.5 MPa, and ~4.5 ± 1.2%, respectively, which are comparable to those of previously examined CNF films (Tables S1–S3) [11,12]. Young’s modulus and tensile strength gradually increased with increasing BNNS content without compromising the elongation. Young’s modulus, tensile strength, and elongation of the 5 wt % BNNS-containing CNF film were ~7.2 ± 0.9 GPa, ~109.5 ± 5.8 MPa, and ~4.5 ± 1.2%, respectively. These Young’s modulus and tensile strength values were approximately 1.52- and 1.19-fold greater, respectively, than those of pristine CNF. In most cases, reinforcing fillers improve the tensile strength but reduce the elongation of composite materials [43,44]. In other words, the reinforced composite becomes stronger but more brittle. The improvement in stiffness without compromising stretchability indicates that the 5 wt % BNNS-containing CNF film is tougher than the pristine film. The good adhesion between BNNS and CNF likely enabled improved toughness. However, the data interpretation is limited to three measurements for each sample.

The tensile properties of the composites are as great as those of engineering plastics, e.g., polycarbonate. Actually, the bio-based plastics, e.g., polylactic acid (PLA) have poorer mechanical properties than the commodity plastics, e.g., PP and PE that are widely used as food-packaging films [45,46]. Along with the poor oxygen barrier, this low mechanical properties of the bio-based plastics is a main reason for the difficulty in commercialization. Thus, this result is encouraging for an increase in the use of bio-based polymeric materials.

### 3.8. In Vitro Cytotoxicity Test of the BNNS-Containing CNF Composite

**Figure 7:**
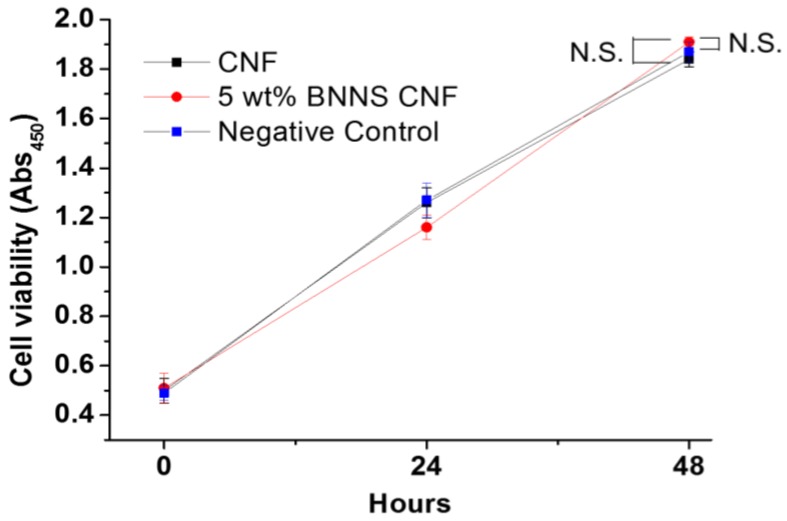
Cell viability tests of MC3T3 proliferation on pristine and 5 wt % BNNS-containing CNF films. The data of triplicate samples represent mean ± deviation. Statistical significance of 0 and 5 wt % BNNS-containing samples and negative control (N.S., not significant, *p* > 0.05; *, *p* < 0.05; unpaired *t*-test).

To examine the cytotoxicity of BNNS to mammalian cells (MC3T3-E1), viable cells on an empty cell culture well were used as a control, and viable cells on pristine and 5 wt % BNNS-containing CNF films were monitored for 48 h via colorimetric assay (Figure 7). The number of viable cells on the pristine and 5 wt % BNNS-containing CNF films gradually increased and became slightly more abundant than those on the empty well over 48 h, probably because the hydroxyl groups of CNF are more absorbable than the polystyrene surface. There was no significant difference in the number of viable cells (*p* > 0.05) with the addition of BNNS, indicating that BNNS exhibited no cytotoxicity towards the MC3T3-E1 cells. However, the side effects of BNNS in humans have not been studied from a long-term perspective. Further testing is required to prove the lack of cytotoxicity of BNNS when BNNS is exposed to food and beverages.

## 4. Conclusions

In summary, the CNF/BNNS composite film exhibited good oxygen-barrier properties and an OTR of <5 cc/m^2^/day, which is suitable for use as a packaging material for meat and cheese. By the simple addition of BNNS particles to the CNF aqueous solution, without modifying the CNF film fabrication process, the resultant film exhibited improved oxygen-barrier and tensile properties. Owing to the synergistic combination of CNF and BNNS, the tensile strength was improved without sacrificing elongation. Finally, the composite film showed no cytotoxicity to MC3T3 cells, indicating the great potential of the prepared film for food packaging.

## Supplementary Materials

The following are available online at http://www.mdpi.com/2073-4360/10/5/501/s1, Figure S1: Dog-bone shape samples for tensile tests, Figure S2: Tensile strain stress curves of 0% BNNS containing CNF films, Figure S3: Tensile strain stress curves of 1% BNNS containing CNF films, Figure S4: Tensile strain stress curves of 3% BNNS containing CNF films, Figure S5: Tensile strain stress curves of 5% BNNS containing CNF films, Table S1: Young’s modulus (GPa) statics from tensile stress strain curves: values, mean, 48 mean absolute deviation of triplicate trials, Table S2: Ultimate tensile strength (MPa) statics from tensile stress strain curves: values, 51 mean, mean absolute deviation of triplicate trials, Table S3: Elongation at break (%) statics from tensile stress strain curves: values, mean, mean 54 absolute deviation of triplicate trials.
