# Supplementary material for: Sustainable Boron Nitride Nanosheet-Reinforced Cellulose Nanofiber Composite Film with Oxygen Barrier without the Cost of Color and Cytotoxicity
Source: Polymers (Basel). 2018 May 5;10(5):501. doi: 10.3390/polym10050501 (PMC6415411; doi:10.3390/polym10050501)
Supplement: Supplementary file 1 [file polymers-10-00501-s001.docx]

Supplementary Materials

Sustainable Boron Nitride Nanosheet-Reinforced Cellulose Nanofiber Composite Film with Oxygen Barrier without the Cost of Color and Cytotoxicity

Hoang-Linh Nguyen^1,2^, Zahid Hanif^1^, Seul-A. Park^1^, Bong Gill Choi^3^, Thang Hong Tran^1,4^, Dong Soo Hwang^2^, Jeyoung Park^1,4,*^, Sung Yeon Hwang^1,4,*^ and Dongyeop X. Oh^1,4,*^

^1^ Research Center for Bio-based chemistry, Korea Research Institute of Chemical Technology (KRICT), Ulsan 44429, Republic of Korea;

^2^ Division of Environmental Science & Engineering, Pohang University of Science and Technology (POSTECH), Pohang 37673, Republic of Korea;

^3^ Department of Chemical Engineering, Kangwon National University, Ganwan-do, Samcheok 25913, Republic of Korea;

^4^ Advanced Materials and Chemical Engineering, University of Science and Technology (UST), Daejeon 34113, Republic of Korea; jypark@krict.re.kr, dongyeop@krict.re.kr

***** Correspondence: [jypark@krict.re.kr](mailto:jypark@krict.re.kr); Tel.: +82-52-241-6315, [crew75@krict.re.kr](mailto:crew75@krict.re.kr); Tel.: +82-52-241-6313, [dongyeop@krict.re.kr](mailto:dongyeop@krict.re.kr); Tel. +82-52-241-6316:


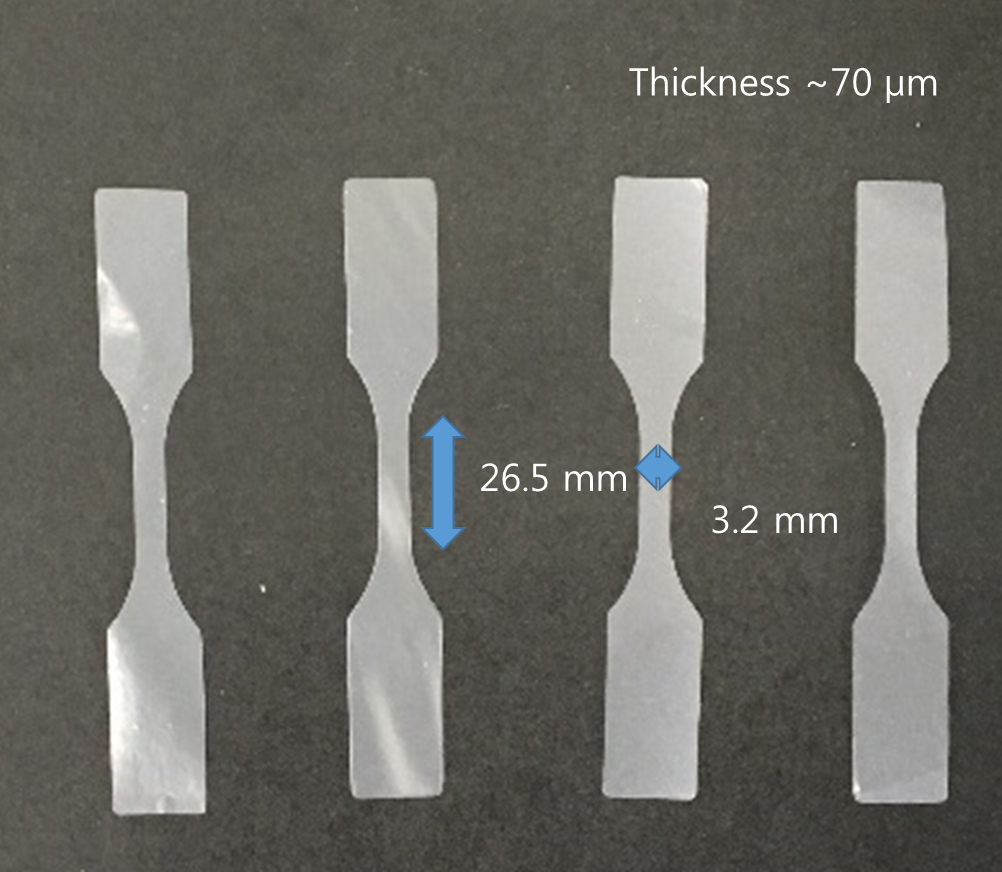


Figure S1. Dog-bone shape samples for tensile tests.

Figure S2. Tensile strain stress curves of 0% BNNS containing CNF films

Figure S3. Tensile strain stress curves of 1% BNNS containing CNF films.

Figure S4. Tensile strain stress curves of 3% BNNS containing CNF films.

Figure S5. Tensile strain stress curves of 5% BNNS containing CNF films.

**Supplementary discussion**

1. Mean

$$n^{-1}\sum_{i=1}^{n} X_{i}$$

2. Mean absolute deviation (MAD)

$$n^{-1}\sum_{i=1}^{n} \left| X \right._{i}-\left. Mean \right\rceil$$

3. Data presentation

The number of data is rounded up to three decimal places.

|  | Results of triplicate samples | | | Mean | Deviation |
| --- | --- | --- | --- | --- | --- |
| 0 wt% | 4.7 | 4.2 | 5.1 | 4.7 | 0.3 |
| 1 wt% | 6.1 | 4.2 | 4.9 | 5.1 | 0.7 |
| 3 wt% | 6.2 | 7.1 | 6.7 | 6.6 | 0.3 |
| 5 wt% | 8.5 | 6.9 | 6.1 | 7.2 | 0.9 |

Table S1. Young’s modulus (GPa) statics from tensile stress strain curves: values, mean, mean absolute deviation of triplicate trials.

|  | Results of triplicate samples | | | Mean | Deviation |
| --- | --- | --- | --- | --- | --- |
| 0 wt% | 81.2 | 99.3 | 83.2 | 88.1 | 7.5 |
| 1 wt% | 81.5 | 93.0 | 98.1 | 91.0 | 6.2 |
| 3 wt% | 82.4 | 104.3 | 96.7 | 94.0 | 8.0 |
| 5 wt% | 118.1 | 106.9 | 103.3 | 109.5 | 5.8 |

Table S2. Ultimate tensile strength (MPa) statics from tensile stress strain curves: values, mean, mean absolute deviation of triplicate trials.

|  | Results of triplicate samples | | | Mean | Deviation |
| --- | --- | --- | --- | --- | --- |
| 0 wt% | 4.8 | 4.2 | 4.5 | 4.5 | 1.2 |
| 1 wt% | 4.0 | 4.9 | 5.9 | 4.9 | 0.9 |
| 3 wt% | 3.6 | 4.1 | 4.9 | 4.2 | 0.6 |
| 5 wt% | 5.9 | 4.0 | 3.2 | 4.5 | 1.2 |

Table S3. Elongation at break (%) statics from tensile stress strain curves: values, mean, mean absolute deviation of triplicate trials.
